# Graft Intra-Articular Remodeling and Bone Incorporation in ACL Reconstruction: The State of the Art and Clinical Implications

**DOI:** 10.3390/jcm11226704

**Published:** 2022-11-12

**Authors:** Lorenzo Moretti, Davide Bizzoca, Giuseppe Danilo Cassano, Nuccio Caringella, Michelangelo Delmedico, Biagio Moretti

**Affiliations:** 1 Orthopaedic and Trauma Unit, Department of Translational Biomedicine and Neuroscience “DiBraiN”, University of Bari “Aldo Moro”, Piazza Giulio Cesare 11, 70120 Bari, Italy; 2 PhD Program in Public Health, Clinical Medicine and Oncology, University of Bari “Aldo Moro”, Piazza Giulio Cesare 11, 70124 Bari, Italy

**Keywords:** anterior cruciate ligament, anterior cruciate ligament reconstruction, graft healing, graft incorporation, graft ligamentization, graft remodeling, return to sport

## Abstract

The knee is one of the most frequently affected joints in sports trauma, and anterior cruciate ligament (ACL) injury and meniscal tears are the most common lesions. ACL reconstruction (ACLR) remains the treatment of choice for patients willing to return to their previous activity. There are different surgical techniques and different possible usable grafts. The graft used for ACLR surgery undergoes a bone incorporation process and an intra-articular remodelling named ligamentization until it reaches characteristics similar to the native ligament. After the first incorporation stage, the remodelling process is divided into an early stage that could last 4 weeks, a proliferative stage that lasts 4 to 12 weeks, and a final stage of ligamentization that could last over 1 year. The period of return to sport (RTS) after ACLR, which is becoming shorter and shorter, can be a high-risk period for athletes due to the risk of graft failure. This systematic review aims to define the phases of the ligamentization process considering graft type and fixation techniques, as well as the graft’s anatomopathological and biomechanical characteristics, to evaluate a criterion-based rehab progression and maximize patient outcomes for an RTS respecting graft biology. The rehabilitative program has to promote and optimize the graft remodelling and incorporation processes; moreover, it has to accommodate physiological graft healing and avoid overloading. An early RTS and noncompliance with the biological characteristics of the graft in the various phases are associated with a high incidence of re-injury.

## 1. Introduction

High sport injury rates have been reported. The knee is one of the most frequently affected joints in sports trauma for its central role in supporting large and rapidly changing external loads during activity. An anterior cruciate ligament (ACL) injury and meniscal injury are the most common knee lesions, and ACL reconstruction (ACLR) remains the treatment of choice in patients willing to return to previous activity.

The graft used for ACLR surgery undergoes a bone tunnel incorporation process and an intra-articular remodelling named “ligamentization” [1,2,3]. The biological integration between the graft and the bone surface has different pathways and timings considering different aspects such as the graft used, fixation technique, morphotype-related stress, and functional requests [1,2,3,4,5,6].

The intra-articular remodelling process produces graft adaptive transformations; however, it does not have the same biological and mechanical properties as native ACL [1,2,3,4,5,6,7]. This results in a direct influence on knee mechanical proprieties and subsequent different rehabilitation and timing of the complete restoration of articular function after ACLR.

This study aims to define the phases of the ligamentization process considering graft type and fixation techniques, as well as the graft’s anatomopathological and biomechanical characteristics, to evaluate a criterion-based rehab progression and maximize patient outcomes for an RTS concerning graft biology.

## 2. Search Strategy

The review was performed according to Preferred Reporting Items for Systematic Reviews and Meta-Analysis (PRISMA) guidelines. The literature search was carried out on Pubmed, Medline, Cochrane Library, and Google Scholar.

The keywords used were as follows: “ACL graft healing”, “ACL graft remodelling”, “ACL graft ligamentization”, “ACL graft incorporation”, and “ACL reconstruction return to play”.

Two reviewers (M.D. and N.C.) independently screened titles, abstracts, and full texts. Discrepancies between the reviews were thoroughly examined on the full-text screen to avoid inadvertent exclusion. At this stage, a consensus debate mediated by a third reviewer (G.D.C.) overcame potential discrepancies.

Of the 5726 studies collected (82 from Medline, 2094 from Pubmed, 739 from Cochrane Library, and 2811 from Google Scholar), 5447 were discarded after reading titles, 128 duplicates were removed, and 90 were discarded after abstract review. The full text of the remaining 61 studies was examined in greater detail. Forty-three studies were finally included in the review.

## 3. Incorporation and Remodeling

According to the definition of a rehabilitative program after surgery and the correct timing for a return to activity after a lesion, it is important to know the graft specifics and biological transformations after ACLR [5,6,7,8,9,10]. The rehabilitative program has to promote and optimize the graft remodelling and incorporation processes; moreover, it has to accommodate physiological graft healing and avoid overloading [1,2,3,4,5,6,7].

The human graft remodelling process could last 1 year. The initial phase is characterized by graft necrosis and reduction in cellularity without vascularization; during the subsequent stages, there is cell proliferation followed by a ligamentization process with comparable acquired proprieties of the native ligament. 

### 3.1. Incorporation

Graft integration depends on its variety, its placement, its way of fixation, and its micro-movements within the bone tunnel. 

There are different techniques for ACL reconstruction and fixation. Some involve the creation of a complete bone tunnel at the tibial or femoral level, while others involve a tibial half tunnel and a femoral half tunnel. Lastly, there are hybrid techniques in which a femoral half tunnel and a complete bone tunnel are created; in the latter, the neoligament is blocked with suspension systems at the femoral level and with an interference screw at the tibial level according to the technique.

The graft could be an autograft taken from the patellar tendon, quadriceps tendon, hamstring tendon, or peroneus tendon, an allograft from human cadavers and animals, or a synthetic graft even if it is destined for revisions. 

The graft taken from the patellar tendon needs 6 weeks to be incorporated; it goes through a necrosis stage followed by bone incorporation [6]. Bone growth to the graft–bone interface occurs at 3 weeks; a delay is produced by the presence of synovial fluid within the tunnel because it produces an inflammatory response and a secretion of metal-protease, which increases tunnel size and leads to a delay in scar tissue deposition and the graft integration process. Moreover, the consequent micro-movements generate more granulation tissue [3,4,5,6,7,8,9,10,11].

Autografts made up of the semitendinosus tendon and/or gracilis tendon, unlike the patellar graft, have a greater incorporation timing of about 8–12 weeks. Even in this case, there is the first stage of mineralization of the tendon surface followed by bone growth at the most external portion of the graft; finally, there is complete graft incorporation within the bone tunnel with the progressive restoration of collagen fibre continuity with bone (sharp-like fibres).

In addition, allografts from semitendinosus and gracilis tendons need 6–9 months for incorporation and a subsequent longer recovery period. Two fundamental aspects are important for the use of allografts: the immunological effect because the implanted graft could be recognized as non-self, and the sterilization system used, with X-rays to avoid infective processes [4,5,6,11].

### 3.2. Remodeling and Physiokinesitherapy

The remodelling process is divided into an early stage that could last 4 weeks, a proliferative stage that lasts 4 to 12 weeks, and a final stage of ligamentization that lasts 12 weeks to 1 year [1].

The determinant aspects for the remodelling processes are the patient (age, smokers or drug users, concomitant presence of chronic pathologies such as diabetes mellitus, and genetically correlated or haematological diseases that can alter microcirculation in the bone or soft tissue), sports activity, mechanical and surgical factors (correct graft positioning, tensioning, and type), pharmacological factors (NSAIDs delay the remodelling process), and, above all, the subsequent rehabilitation program [7].

#### 3.2.1. Early Stage—Degeneration

The first stage (early stage) constitutes graft degeneration. Fibroblast necrosis is reported in the middle of the graft with a subsequent inflammatory response. Cellular necrosis produces the release of cytokines and chemokines as chemotactic factors for host cells, resulting in stimulation of the neoligament as a scaffold.

At the same time, those cytokines trigger a growth factor cascade that initiates the next stage [9,12,13,14,15,16,17].

The cellular invasion begins at the outer part of the graft between the first and second weeks after surgery; cells derived from the native residual ACL, synovial fluid, and bone marrow elements are released during the bone perforation. During this stage, there is no revascularization. 

A study on rabbits evaluated, 6 days after ACL reconstruction surgery, the marked proline capture on the patellar tendon graft; after 48 h, there was a major marker deposition on the graft surface, demonstrating the presence of active cells. Furthermore, there was no marker deposition to the inner part of the graft with the prevalence of necrosis. This first stage is called the “degeneration stage” [14]. Moreover, an initial degeneration of graft myxoid components is observed but the collagen structure is maintained; this is responsible for the slow decrease in mechanical proprieties [1,9,13,14,15,16].

The major risk during the initial stage is connected to the lack of graft incorporation within the tunnel and graft pull-out [18].

Rehabilitation during this first stage should avoid the tensioning of the new ligament, even if the load is important to promote remodelling [19]; weight-bearing may be delayed in those with concomitant articular cartilage repair or meniscus repair. Rehabilitation during the 4 weeks after surgery is important to support graft incorporation and reduce the pain and inflammatory response (rest, ice, compression, and elevation protocol) because they impede quadriceps motor firing patterns and, upon gaining a range of motion, restore it; specifically, knee extension should be recuperated as soon as possible, as recovering the extension reduces the risk of intercondylar scar tissue accumulation. Shelboune et al. found that even small losses of 3/5° of extension can significantly affect outcomes after ACLR [20].

Flexion recovery must happen quickly and gradually with passive and active exercises: during the first 2 weeks, 90° of knee flexion should be recovered, followed by 120° in the third week, and full flexion in the fourth week.

Furthermore, quadricipital musculature should be strengthened; allowed exercises are isometric quadricipital contractions at 0°, 60°, and 90°, high saddle bicycle with low resistance, hell slides, straight leg raise, closed kinetic chain leg presses, and open kinetic chain extensions [21,22].

#### 3.2.2. Proliferative Stage

The second stage (proliferative stage) from the 4th to 12th week is dominated by elevated cellular activity and important extracellular matrix changes, which start during the earliest period after surgery. At this moment the graft has poor mechanical proprieties. 

Based on animal studies, we can provide two subphases at this stage: a proliferative subphase, characterized by cellular proliferation and reabsorption of the collagen matrix for the deposition of a new one with a vascularization-correlated process; a replicative subphase representing biochemical and histological modifications of the extracellular matrix (increase in collagen type III, increase in fibronectin, increase in diameter of collagen fibres, and increase in GAG content) to produce the recovered mechanical graft properties.

Evaluation in humans has shown reduced remodelling with partial substitution of fibres from cells and reduced necrosis. Vascularization is reduced and related to collagen destruction [23].

Scheffler et al. [9] reviewed cellular activity in grafts at 6 weeks after surgery and evaluated cell clusters in an animal model at the perimeter of the graft, with large acellular areas remaining in the graft’s centre. On the other hand, Rougraff et al. [24] found areas of hypercellularity and very plump nuclear morphology in patients who underwent second-look arthroscopy and biopsy 3 to 8 weeks after anterior cruciate ligament (ACL) reconstruction using an autogenous patellar tendon graft. These studies showed a collagen matrix disorganization and an increase in cellularity compared to the native LCA, even if present mainly in the areas of vascular invasion; this could be found in both animal and human models.

The rehabilitation program during this period accepts load to stimulate the production of cellular and extracellular components, preserving graft stability. The rehabilitation goal should be a normal gait with complete knee flexion and extension; the gait could also be altered as a result of compensatory techniques used when the knee was sore immediately after surgery. This change in gait may be more subconscious at this point. It may be beneficial to use a mirror so that the athlete can see themselves and their irregular gait pattern. Another exercise to perform is a high-stepping gait cycle with a mirror, which can be used to make the patient understand the degree of hip and knee flexion that can be reached without feeling pain.

Isometric muscle contractions can be replaced by isotonic muscle contractions to improve graft strength during the incorporation process and due to necrosis being succeeded by revascularization. Quadriceps strengthening can begin with wall slides progressing to mini-squats or progressive resistive leg press exercises; at this time, enough soft-tissue healing of the hamstrings should allow tolerance to perform gentle hamstring and gastrocnemius and soleus flexibility exercises. Squats with a closed kinetic chain should be performed on a solid surface before progressing to an unstable or labile surface. The athlete is instructed to flex the knees to approximately 25–30° and sustain the position to produce hamstring and quadriceps co-contraction. Squats may be performed on a tilt board that is tilted in either the medial/lateral or the anterior/posterior directions. Particular attention must also be paid to proprioceptive exercises.

Lunges in multiple directions can gradually be included in the physiotherapy program. Higher-level lunges can be incorporated by lunging with a rotation, lunging onto a labile surface, or lunging with perturbation. When these lunges are tolerated, the patient’s attention can be focused on throwing or catching a ball indicative of the practised sport (basketball, football, etc.). This phase is marked by the advancement of the patient from tolerating simple functional activities to more advanced activities, enabling them to progress to complete leisure or sporting activities. They should have enough strength and balance by the end of 12 weeks to advance to regulated individual sports and leisure activities. This does not suggest that they will be entirely autonomous once they resume sports, but it does imply that they will be able to participate in supervised individual functional sports activities [21,22].

#### 3.2.3. Ligamentization Stage

The third stage (ligamentization stage) starts 12 weeks after surgery. At this point, the graft undergoes adaptive remodelling to achieve the mechanical properties and structure of the native ACL. The native ACL is composed of aligned collagen fibres with different diameters. After the second stage, the graft is composed of disorganized fibres in space and diameters. Animal models show the normalized composition of cells in the graft at 3–6 months after surgery; vascularization is remodelled as the native ACL 6–12 months after surgery, and fibres reorganize themselves 6 to 12 months after surgery, despite their diameter showing a unimodal pattern. There is an increased production of collagen type III, which has fewer mechanical properties than collagen type I typical of native ACL [2,3,4,5,6,7,8,9]. Several studies on human models have tried to define the timing for vascularization as between 6 and 12 months, for collagen fibre organization as 6 months [8], for crosslink bonds between collagen fibres as 1 year [25], and end of the remodelling process of collagen fibres at almost 2 years [23,24,25,26,27].

After 2 years after surgery, the end of the revascularization process matches with a homogeneous graft MRI signal close to the native ACL [28].

At 6 months after surgery, a graft implanted in human shows a reduction in collagen content compared to the original ligament, the interfibrillar spaces are occupied by an extracellular matrix, and clusters of fibrils are arranged parallel to the fibres; the presence of elastic fibres and similar-function macrophage cells, tenocyte cells, and fibroblasts is also observed.

The reconstructed ACL is constituted by large-diameter collagen fibres similar to normal ligaments, along with small clusters of fibres of low diameter 12 months after surgery. Fibrils have a multidirectional organization which is parallel, oblique, and perpendicular to the main neoligament axis. The elastic system is well represented by elastic and oxalic fibres. An increased volume of the rough endoplasmic reticulum within cells demonstrates important protein synthesis [8,24].

The new ligament is very similar to the native ACL after 24 months; the small collagen fibrils are organized in more distended and closely packed fibres with a multidirectional organization, the interfibrillar space is reduced, and there is a small amount of extracellular matrix. Cells and the elastic system 2 years after surgery are almost the same as those observed at 12 months [25,26].

After 4 years after surgery, the graft is even more similar in collagen and fibres space orientation to the native ACL, with a further reduction in interfibrillar gaps and an increase in the elastic system. At 10 years after surgery, fibre arrangement is close to that observed at 24 months. The tendon is architecturally transformed almost completely into a native ACL; however, some fibres of the original tendon remain inside the graft [2,23].

The end of the ligamentization process was indicated at 3 years after surgery by Rougraf et al. [24], at 1 year by Abe et al. [27] and Falconiero et al. [8], and 2 years by Sanchez et al. [29], even though the autograft completes the transformation process at 10 years after implant; the persistent mechanical stress on the graft helps it to arrange the fibres similarly to native ACL. 

Even the evoked potentials (EPs) become similar between patients subjected to LCA reconstruction and naïve patients, with a negative correlation between EP voltage and knee instability [30,31].

Moreover, there is a difference between animal and human models; reinnervation is observed in rabbit grafts after ACL reconstruction [32] but is absent in humans. A study with the use of silver immunostaining demonstrated reinnervation without neuropeptide immune reaction, explaining the presence of nerve structures that are not metabolically active [33].

A low level of evidence is available for the reinnervation graft process; proprioceptive improvements after surgery are probably connected to central nervous system modifications or neuroplasticity. Patients with reconstructed ACLs show an increase in cerebral activity in the contralateral motor cortex, ipsilateral angular gyrus, and somatosensory cortex, whereas reduced activity was recorded in the ipsilateral motor cortex and cerebellar vermis [34,35,36,37,38,39,40,41,42].

Biomechanical proprieties reached 1 year after surgery can be considered as 50–60% of those types of the native ACL [2]. 

However, in recent years, various ACL repair techniques have been experimented with, providing excellent outcomes [43].

During this third phase, the patient trains for a gradual return to full sports activity. If they pass the clinical examination and have no swelling or discomfort while undertaking daily activities, have a full range of motion, and have no instability, they can begin jogging in a straight line.

When tolerated, bilateral plyometric exercises requiring a stretch/shortening period may be progressed to unilateral exercises. Exercises that stress single-limb postural control are given first on level ground and then progressed to an unstable labile surface. Improving the balance of a single limb can be initiated by maintaining the position of only one limb on a flat and level surface while moving the contralateral extremity through classic movement patterns such as flexion/extension and abduction/adduction. Once this has been demonstrated, inclined planes and proprioceptive platforms can be used. Running and jumping exercises, strength and endurance exercises, gait exercises, and valgus overload of the knee can be implemented.

Exercises for the trunk and hips are recommended because most advanced activities need core stability to retain the centre of mass, balance, and postural control. The ability to effectively regulate the body’s centre of mass in response to forces produced by distal body parts during athletic competition is linked to core trunk training and stability [35].

## 4. Return to Sport

Return to playing does not mean full participation in competitive events; athletes are advised to resume practice activities and begin to prepare for competitive play gradually.

The mean absence until the first match appearance after undergoing ACL reconstruction was 237.5 ± 76.1 days in Walden’s study [36], 232 ± 135 days in Roi’s study [37], and 192 ± 85 days in Faude’s [38] study.

However, return to match play is influenced by several nonmedical factors, such as coach selection, off-season holidays, and playing position.

An early return to sport, <160 days, is associated with a high incidence of reinjury considering ligamentization and loss of physical performance timing.

For a safe return to sport, we should consider the van Grinsven criteria [39] (no pain or swelling, complete ROM, quadriceps and hamstring strength >85%, the difference in hamstring/quadriceps strength ratio <15%, hop tests >85% compared to the contralateral side, exercises of the previous week carried out properly, and tolerance of sport-specific activities and agility training with maximal duration and speed), Adams criteria [40,41] (limb symmetry index >90%, 90% or greater on all hop tests, and 90% or greater on KOS-ADL), and psychological evaluation (the Tampa Scale of Kinesiophobia (TSK) that evaluates fear of movement and fear of a new injury, as well as the ACL Return to Sport after Injury (ACL-RSI) questionnaire that evaluates frustration, fears, and security, emotional response to injury, motivation, and athlete confidence).

Hadley et al. showed that, for a safe RTS, patients must pass a checklist indicated in their work. Patients who successfully passed the checklist before returning to play experienced a significantly lower incidence of ipsilateral ACL injury as compared with patients who did not pass the checklist [42].

## 5. Conclusions

The graft used for ACLR surgery undergoes a bone incorporation process and ligamentization process until it reaches characteristics similar to the native ligament.

It is important to know the graft specifics and biological transformations after ACLR for the correct timing to return to activity after injury. The rehabilitative program has to promote and optimize graft remodelling and incorporation processes; moreover, it has to accommodate physiological graft healing and avoid overloading. An early RTS and noncompliance with the biological characteristics of the graft in the various phases are associated with a high incidence of re-injury.

Further studies are needed to evaluate whether the graft’s microstructural characteristics and cellularity change after repair.

## Data Availability

Not applicable.
